# Aspirin alleviates hepatic fibrosis by suppressing hepatic stellate cells activation via the TLR4/NF-κB pathway

**DOI:** 10.18632/aging.103002

**Published:** 2020-04-13

**Authors:** Yan Liu, Li Nong, Yuxian Jia, Aihua Tan, Lixia Duan, Yongkui Lu, Jinmin Zhao

**Affiliations:** 1 The Fifth Department of Chemotherapy, Affiliated Tumor Hospital of Guangxi Medical University, Nanning 530021, Guangxi Zhuang Autonomous Region, China; 2 Guangxi Key laboratory of Regenerative Medicine, Guangxi Medical University, Nanning 530021, Guangxi Zhuang Autonomous Region, China; 3 Division of Spinal Surgery, The First Affiliated Hospital of Guangxi Medical University, Nanning 530021, Guangxi Zhuang Autonomous Region, China

**Keywords:** aspirin, hepatic fibrosis, inflammation, hepatic stellate cells

## Abstract

Hepatic fibrosis arises from a sustained wound-healing response to chronic liver injury. Because the occurrence and development of hepatic fibrosis is always associated with chronic inflammation, controlling inflammation within the liver may be an effective means of controlling the development and progression of hepatic fibrosis. Aspirin is a non-steroidal anti-inflammatory drug used to relieve both inflammatory symptoms and pain. The results of our study showed that aspirin significantly attenuated hepatic inflammation and fibrosis. Aspirin effectively inhibited the activation and proliferation of hepatic stellate cells (HSCs), which led to downregulation of inflammatory factors, including IL-6 and TNF-α in those cells. Aspirin also downregulated expression of Toll-like receptor-4 (TLR4) on HSCs, as well as its downstream mediators, MyD88 and NF-κB. The results of our study demonstrate aspirin’s potential to inhibit the development of hepatic fibrosis and the molecular mechanism by which it acts. They suggest aspirin may be an effective therapeutic agent for the treatment of hepatic fibrosis.

## INTRODUCTION

Hepatic fibrosis is a consequence of a sustained wound-healing response to chronic liver damage. Progressive hepatic fibrosis leads to cirrhosis and hepatocellular carcinoma [1]. Because chronic inflammation with the liver is associated with the occurrence of hepatic fibrosis, controlling inflammation could be an effective strategy for controlling the development of hepatic fibrosis. In that regard, aspirin is a traditional non-steroidal anti-inflammatory drugs that is frequently prescribed to relieve pain and attenuate inflammatory symptoms. Moreover, it has been reported that aspirin prevents the development of fibrosis [2, 3], though the mechanism remains unclear.

Hepatic stellate cells (HSCs) are reportedly a critical contributor to fibrogenesis within the liver [4]. Indeed, HSC activation may be the earliest event underlying hepatic fibrogenesis. Toll-like receptors (TLRs) recognize pathogen-associated molecular patterns and play an important role in leading inflammatory responses [5]. TLR4 is constitutively expressed in multiple liver cell types, including liver vascular endothelial cells, Kupffer cells and HSCs [6]. The binding of lipopolysaccharide (LPS) to TLR4 within the liver initiates an inflammatory response that results in inflammation-associated liver damage [7–9]. In HSCs, TLR4-mediated hepatic fibrosis appears to depend on transforming growth factor-β (TGF-β)-dependent collagen production [9]. In the present study, we examined the effect of aspirin on carbon tetrachloride (CCl_4_)-induced hepatic fibrosis and explored the potential mechanism.

## RESULTS

### Aspirin attenuates hepatic fibrosis and liver inflammation in rat

**Figure 1:**
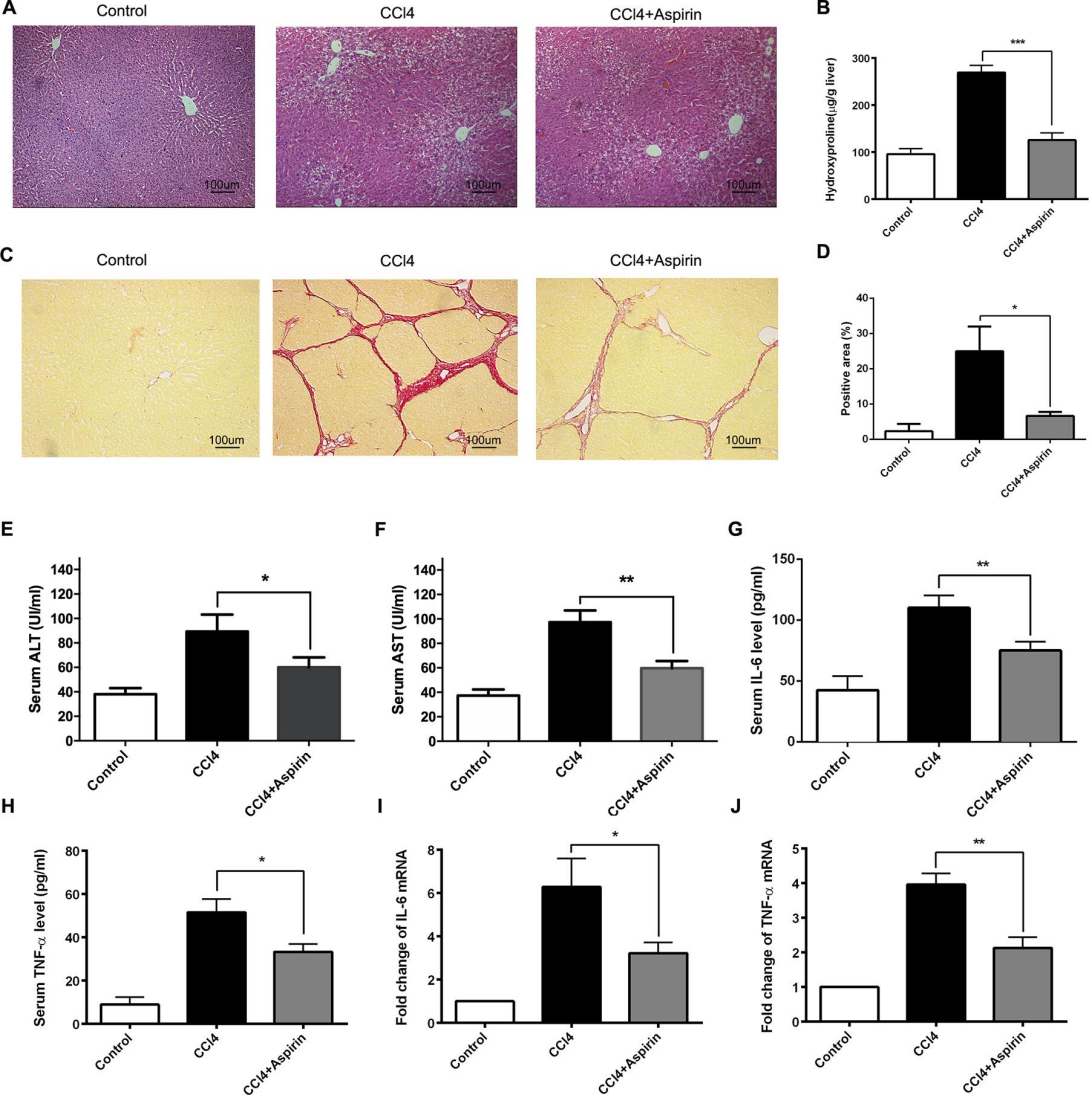
**Aspirin rehabilitated CCl4-induced liver fibrosis and inflammation in rat.** (**A**) Liver fibrosis was detected 6 weeks after CCl4 treatment by HE. (×200; scale bar: 100μm) (**B**) Hepatic hydroxyproline content was measured 6 weeks after CCl4 treatment. (**C**, **D**) Hepatic fibrosis was examined by Sirius red staining. (×200; scale bar: 100μm) (**E**, **F**) The AST and ALT were detected to assess the liver function. (**G**–**J**) The levels of inflammatory cytokines (IL-6 and TNF-α) in serum and liver tissues were measured by ELISA and real-time PCR. ^*^P<0.05, ^**^P<0.01.

To investigate the role of aspirin in hepatic fibrosis, we analyzed liver sections from CCl_4_-induced rats, with and without aspirin treatment. The histological status of the liver was assessed by using hematoxylin-eosin (HE) staining. The result showed that compared with control group, aspirin significantly reduced the necrotic area and the number of inflammatory cells in liver tissue (Figure 1A). Furthermore, aspirin treatment (100 mg/kg) effectively decreased hydroxyproline levels and collagen accumulation as compared to untreated controls (Figure 1B–1D). We also observed that the activities of the liver enzymes aspartate aminotransferase (AST) and alanine aminotransferase (ALT) were lower in the aspirin group than control group, indicating improved liver function (Figure 1E and F). Correspondingly, serum and hepatic levels of both IL-6 and TNF-α were lower in the aspirin group than control group (Figure 1G–1J), suggesting aspirin treatment led to downregulation of inflammatory cytokines. These findings suggest that aspirin treatment reduces hepatic inflammation and damage in this rat model.

### Aspirin treatment leads to downregulation of profibrogenic associated mediators and TLR4

**Figure 2:**
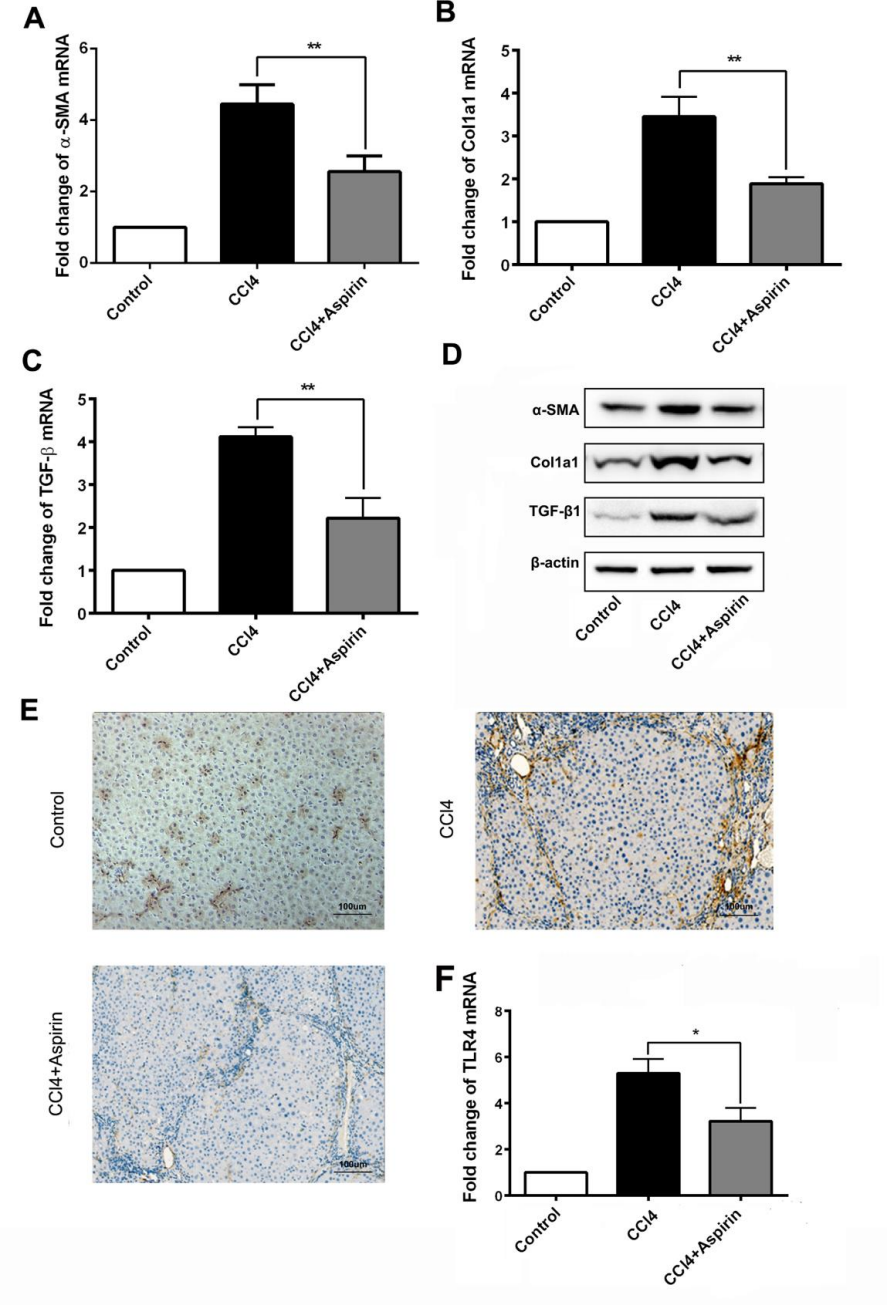
**Aspirin reduces expression of profibrogenic associated mediators and TLR4.** (**A**–**C**) mRNA expression of early markers of fibrogenesis including α-SMA, collagen-a1 and TGF-β1 was detected by real-time PCR. (**D**) Western blot was employed to detect the expression ofα-SMA, collagen-a1 and TGF-β1 in liver tissues. (**E**, **F**) TLR4 expression was tested by real-time PCR and immunochemistry analysis. (×200; scale bar: 100μm) ^*^P<0.05, ^**^P<0.01.

We employed real-time PCR and western blot to observe the effect of aspirin on profibrogenic associated factors and TLR4 in liver tissues. We found that with aspirin administration, the expression of the profibrogenic mediators collagen-a1(I) (encoded by *Col1a1*), α-SMA and TGF-β1, was decreased (Figure 2A to 2D). Also reduced was the mRNA and protein expression of the pattern recognition receptor TLR4 (Figure 2E and 2F), which is a central event in the progression of hepatic fibrosis [9, 10]. These results suggest that aspirin effectively reduces hepatic expression of profibrogenic mediators and TLR4.

### Inhibitory effect of aspirin on HSC activation

**Figure 3:**
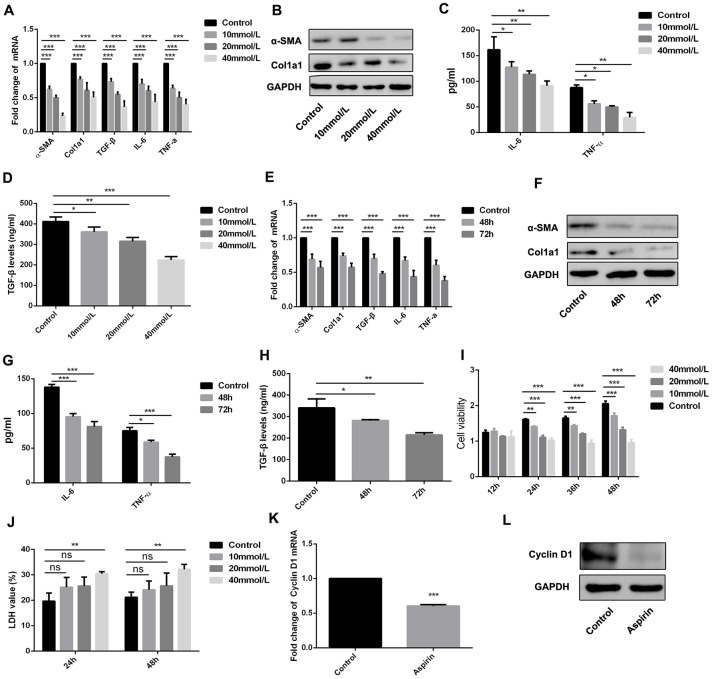
**Aspirin inhibited the activation and proliferation of hepatic stellate cells.** (**A**, **B**) Real-time PCR and western blot was employed to examine the expression of α-SMA, collagen-a1, TGF-β, IL-6 and TNF-α in the LPS-treated HSCs with disposure of 0, 10, 20, 40mmol/L. (**C**, **D**) ELISA assay was used to examine the expression of TGF-β, IL-6 and TNF-α in the LPS-treated HSCs with disposure of 0, 10, 20, 40mmol/L. (**E**, **F**) Real-time PCR and western blot was employed to examine the expression of α-SMA, collagen-a1, TGF-β, IL-6 and TNF-α in the LPS-treated HSCs with disposure of 40mmol/L at 48 and 72h. (**G**, **H**) ELISA assay was used to examine the expression of TGF-β, IL-6 and TNF-α in the LPS-treated HSCs with disposure of 40mmol/L at 48 and 72h. (**I**) CCK-8 assays were performed to examine the proliferation of LPS activated-HSCs with aspirin treatment. (**J**) LDH assay was performed to detect the cell viability of LPS activated-HSCs with aspirin treatment. (**K**, **L**) The expression of Cyclin D1 was detected by real-time PCR and western blot. ^*^P<0.05, ^**^P<0.01, ^***^P<0.001.

HSCs are reported to be a critical cell population contributing to fibrogenesis in the liver, and LPS plays a key role in activating HSCs [11, 12]. We therefore examined the effect of aspirin on LPS-induced expression of inflammatory and fibrogenic genes in HSCs. Our results show that aspirin administration led to downregulation of α-SMA, collagen I, and TGF-β1 in LPS-activated HSCs. In addition, aspirin also inhibited expression of both IL-6 and TNF-α. These effects of aspirin were both dose- and time-dependent (Figure 3A–3H).

Using CCK-8 assays, we also observed that aspirin dose- and time-dependently suppressed HSC proliferation as compared to control groups (Figure 3I). We also employed LDH assay to examine the role of aspirin on the cell viability of HSC. As shown in figure 3J, aspirin on 40 mmol/L could effectively inhibit the cell viability of HSC. Moreover, real-time PCR analysis of Cyclin D1 expression showed that aspirin treatment effectively decreased Cyclin D1 expression in HSCs within 48 h (Figure 3K and 3L). These results confirm that aspirin exerts inhibitory effect on both HSC activation and proliferation.

### Aspirin inhibits MyD88 and NF-κB in HSCs

**Figure 4:**
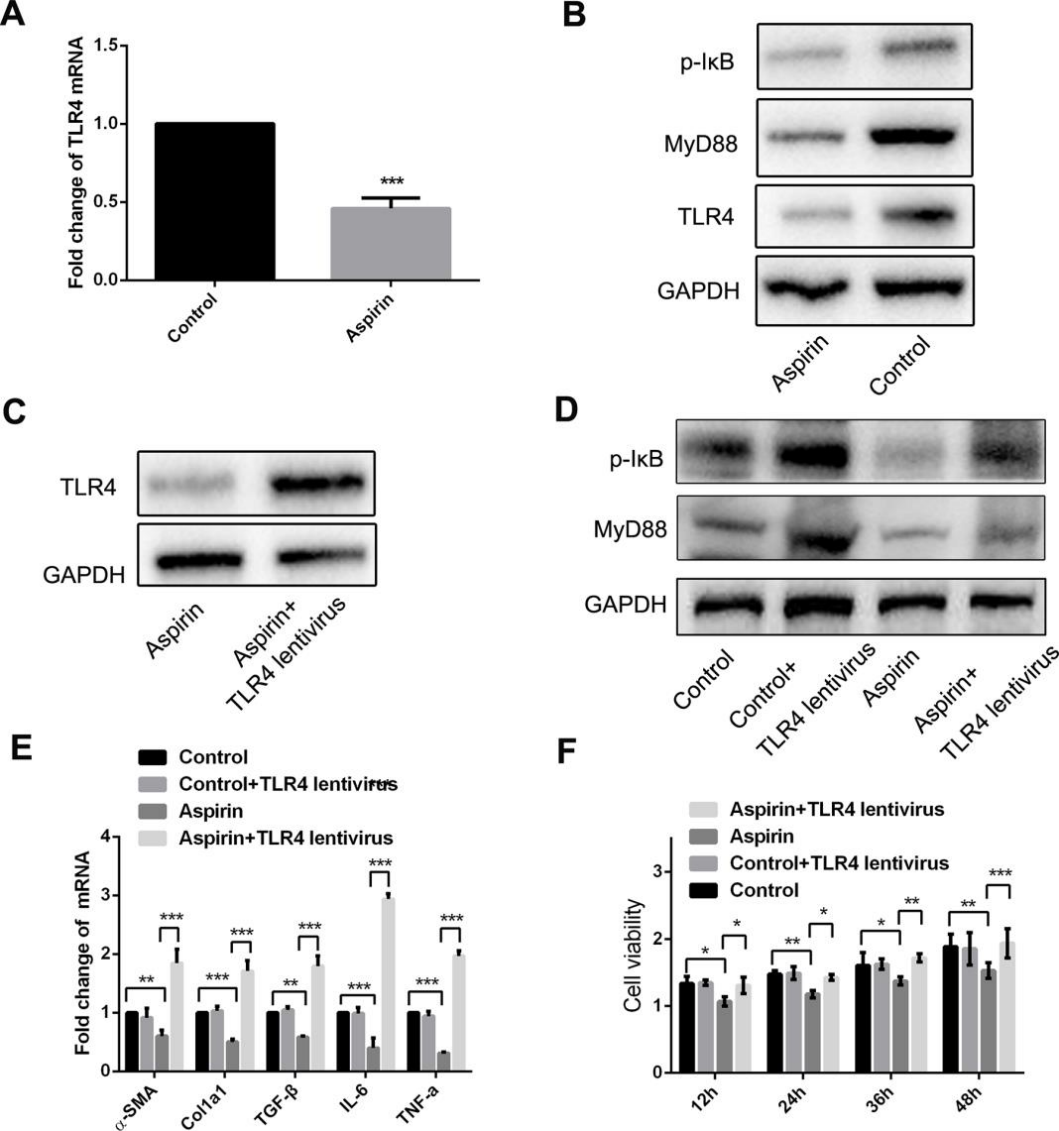
**MyD88 and NF-κB signaling mediated the inhibitory effect of HSCs activation and proliferation by aspirin.** (**A**) Real-time PCR was performed to analysis the expression of TLR4 in LPS-pretreated HSCs with or without aspirin(40mmol/L) disposure. (**B**) Western blot was used to examine the protein expression of TLR4, MyD88 and p-IκB in LPS-activated HSCs treated with aspirin. (**C**) TLR4 expression was detected in LPS-activated HSCs treated with aspirin and TLR4 lentivirus at 48h. (**D**) MyD88 and p-IκB in LPS-activated HSCs treated with aspirin and TLR4 lentivirus at 48h were measured by western blot. (**E**) Real-time PCR was employed to examine the expression of α-SMA, collagen-a1, TGF-β, IL-6 and TNF-α in activated HSCs treated with aspirin (40mmol/L) and TLR4 lentivirus. (**F**) CCK-8 assays were performed to examine the proliferation of activated HSCs treated with aspirin and TLR4 lentivirus HSCs. ^*^P<0.05, ^**^P<0.01, ^***^P<0.001.

The results summarized above show that aspirin inhibits TLR4 expression (Figure 2E and 2F). Furthermore, when we used real-time PCR and western blot to examine expression of components in the TLR4 signaling pathway in LPS-activated HSCs, we found that in addition to TLR4 itself, aspirin administration led to the downregulation of myeloid differentiation factor 88 (MyD88) and p-IκB within 48 h (Figure 4A and 4B). Then we investigate the role of MyD88-dependent NF-κB signaling in the activation and proliferation of HSCs. We found that by using a lentivirus vector to overexpress TLR4 in HSCs, we could reverse the aspirin-induced suppression of MyD88 and p-IκB (Figure 4C and 4D), thereby inducing HSC activation and proliferation (Figure 4E and 4F). These results demonstrate that MyD88-associated NF-κB signaling contributes to the reversal of aspirin’s inhibitory effects on HSC activation and proliferation. This confirms the role of the TLR4/NF-κB pathway in mediating HSC activation and hepatic fibrosis.

## DISCUSSION

Hepatic fibrosis arises from wound healing in response to chronic inflammation within the liver [13, 14]. Recently, it was reported that aspirin administration effectively attenuates hepatic fibrosis [15]. Moreover, we observed in the present study that HSC activation and proliferation mediated via the TLR4 signaling pathway were blocked by aspirin.

The occurrence and progression of hepatic fibrosis is tightly associated with chronic hepatic inflammation not only in experimental models of fibrogenesis, but also in virtually all individuals with liver disease [16, 17]. In the present study, aspirin was used to treat a rat model of hepatic fibrosis. The results showed that CCl_4_ administration induced hepatic fibrosis, which was characterized by increased extracellular matrix (ECM) deposition and hydroxyproline content within the liver, and that aspirin reduced hepatic levels of both ECM and hydroxyproline. In addition, aspirin also exerted anti-inflammatory effects, reducing expression of the inflammatory cytokines IL-6 and TNF-α, and antifibrogenic effects, reducing expression of α-SMA, TGF-β1 and collagen I. Thus, aspirin appears to inhibit both inflammation and fibrogenesis during CCl_4_-induced liver damage.

HSC activation is a key step in the pathogenesis of hepatic fibrosis [18]. Consistent with other antifibrotic effects, aspirin also suppressed HSC activation and proliferation. The pattern recognition receptor TLR4 and complements play important roles in the regulation of inflammation, HSC activation and liver fibrosis [8]. We therefore hypothesized that the antifibrotic effects of aspirin may be mediated through suppression of the TLR4 signaling pathway. Consistent with our hypothesis, TLR4 levels were downregulated in aspirin-treated rats.

TLR4 is one of the most important receptors binding LPS, and the MyD88 signaling pathway is one of the most important pathways leading to activation of NF-κB, which ultimately leads to upregulation of the inflammatory cytokines IL-6 and TNF-α [5, 19]. In addition, NF-κB activation increases levels of TGF-β1, which can enhance the survival and proliferation of activated HSCs [20, 21]. In the present study, we found that aspirin inhibited expression of both MyD88 and p-IκB, which means that aspirin blocks the activation and proliferation of HSCs through inhibition of MyD88-dependent NF-κB signaling.

Collectively, the results of our study demonstrate aspirin suppresses hepatic fibrosis by exerting various anti-inflammatory effect that inhibit signaling in the TLR4 pathway, which in turn leads to inhibition of HSC activation and proliferation. This suggests aspirin may be an effective therapeutic agent for the treatment of hepatic fibrosis.

## MATERIALS AND METHODS

### Animals model

Sprague-Dawley (SD) rats (male, 180 ± 15g) were purchased from the Experimental Animal Center of Guangxi Medical University, China. The rats were fed a standard diet and acclimated in a quiet quarantine room for 1 week before the experiments. The rats received subcutaneous injections of CCl_4_ (1 mL/kg, Sigma-Aldrich) diluted 2:3 in olive oil twice weekly for 6 weeks (9 rats in each groups). All animal experiments were approved by the Animal Care and Experimentation Committee of Guangxi Medical University. Animal experimentation methods were carried out in accordance with the approved guidelines.

### Histological staining

Liver tissues were fixed with 10% neutral-buffered formalin and then embedded in paraffin. Paraffin embedded liver tissues were sectioned to a thickness of approximately 4 μm and stained with HE following a standard protocol. To observe collagen deposition, sections were stained with Sirius red and examined under a microscope.

### Measurement of hepatic hydroxyproline content

Total hepatic hydroxyproline levels were determined in liver tissue hydrolysates as described previously [22].

### Immunohistochemical staining

Paraffin embedded liver tissue sections (4 μm thick) were mounted on 3-aminopropyl-triethoxy-silane-coated slides and dewaxed and rehydrated with freshly distilled water. After blocking endogenous peroxidase for 20 min, antigen retrieval was carried out using citrate buffer. The sections were then incubated with primary antibodies overnight at 4°C, washed with PBS, incubated with biotinylated secondary antibody at 37°C for 30 min then streptavidin-peroxidase, and stained with diaminobenzidine. Hematoxylin was used to stain cell nuclei, after which and the sections were dehydrated and mounted for microscopy.

### Enzyme-linked immunosorbent assay (ELISA)

ELISAs were performed using commercial TNF-α, IL-6 and TGF-β ELISA kits (R&D Systems, Minneapolis, MN). All assays were performed in duplicate, and readings were compared with standard curves constructed using standard proteins provided with the kit. Means and standard deviations of concentrations in triplicate samples were compared using a t-test.

### Real-time PCR

Total mRNA was extracted from samples using Trizol Reagent (Invitrogen, Carlsbad, CA, USA). cDNA was then synthesized from 2 μg of total RNA using MMLV reverse transcriptase (Promega, WI, USA) and oligo dT18-primers. Two-microliter aliquots of cDNA were used for PCR amplification. Real-time PCR was performed in triplicate using a SYBR PrimeScript RT-PCR Kit to measure TLR4, TNF-α, IL-6, TGF-β1, α-SMA, Col1A1, Cyclin D1 and GAPDH mRNA. (Takara, China). Levels of sample mRNA were normalized to endogenous GAPDH mRNA. The thermocycling protocol entailed an initial hold at 50°C for 2 min and then 95°C for 10 min followed by 40 cycles of 95°C for 15 s and 60°C for 60 s. The data were collected and quantitatively analyzed using a Mx4000 system (Stratagene, La Jolla, CA). Levels of mRNA expression are presented as the fold change relative to an untreated control.

### Cell culture

Rat hepatic stellate cell line, HSC-T6 were cultured in Dulbecco’s modified Eagle’s medium supplemented with 10% fetal bovine serum (FBS), 100 μg/ml penicillin and 100 μg/ml streptomycin at 37°C under 5% CO_2_. HSCs were activated by exposure to LPS (Sigma, 100 ng/ml). Cells were treated with aspirin at concentrations of 10, 20 or 40 mmol/L for 48 or 72 h.

### Cell counting Kit-8 (CCK-8) assay

HSCs (5×10^3^/well) were plated in 96-well plates and treated with aspirin as described above. The cell viability was then measured using 10% cell counting Kit-8 (CCK-8) assays (g4103, Service Bio, China) according manufacturer’s instructions.

### LDH assay

To confirm the effects of aspirin on cell viability, LDH activity assay was conducted with an LDH kit (Jiancheng Bio-engineering Institute, Nanjing, China), according to the manufacturer's instructions.

### Western blot analysis

Cells were washed in PBS solution, after which proteins were extracted using an established protocol. The proteins were then mixed with Laemmli sample buffer, heated at 65°C for 10 min, loaded (20 μg for each sample), separated by sodium dodecyl sulfate-polyacrylamide gel (7.5%) electrophoresis under denaturing conditions, and electroblotted onto nitrocellulose membranes. The membranes were blocked by incubation in blocking buffer (1% BSA in Tris-buffered saline-0.1% Tween 20), then incubated with primary antibodies, washed, and incubated with anti-rabbit peroxidase-conjugated secondary antibody (1:10,000; Sigma). Immunoblots were developed using a BeyoECL (Beyotime) and Tanon 5200 system. The primary antibodies were as followed: α-SMA (Novus, 1:500), Col1a1 (Abcam, 1:1000), TGF-β (Abcam, 1:500), Cyclin D1 (Abcam, 1:200) p-IκB (Santa crus, 1:200), MyD88 (Abcam, 1:500), TLR4 (Abcam, 1:300).

### Statistical analysis

Statistical analysis of the data was done by using GraphPad Prism 6 (GraphPad Software). Student’s t-test was used to compare mean values between two groups. Comparisons between three or more groups were made using the one-way analysis of variance followed by Dunnett’s post hoc test. Final values are expressed as mean ±standard deviation (SD). Values of P<0.05 were considered statistically significant.
